# Natural cloud brightening enhanced by marine primary organic aerosols

**DOI:** 10.1093/nsr/nwag604

**Published:** 2026-09-17

**Authors:** Wei Xu, Baihua Chen, Lin Wang, Keran Zhang, Lu Lei, Kirsten Nicole Fossum, Emmanuel Chevassus, Liyuan Zhou, Haobin Zhong, Chunshui Lin, Yanan Zhan, Jingye Ren, Dan Dan Huang, Ruqian Miao, Darius Ceburnis, Ru-Jin Huang, Colin O’Dowd, Jurgita Ovadnevaite

**Affiliations:** State Key Laboratory of Advanced Environmental Technology, Institute of Urban Environment, Chinese Academy of Sciences, Xiamen 361021, China; State Key Laboratory of Advanced Environmental Technology, Institute of Urban Environment, Chinese Academy of Sciences, Xiamen 361021, China; State Key Laboratory of Advanced Environmental Technology, Institute of Urban Environment, Chinese Academy of Sciences, Xiamen 361021, China; State Key Laboratory of Advanced Environmental Technology, Institute of Urban Environment, Chinese Academy of Sciences, Xiamen 361021, China; School of Natural Sciences, Centre for Climate & Air Pollution Studies, Ryan Institute, University of Galway, Galway H91 TK33, Ireland; School of Natural Sciences, Centre for Climate & Air Pollution Studies, Ryan Institute, University of Galway, Galway H91 TK33, Ireland; School of Natural Sciences, Centre for Climate & Air Pollution Studies, Ryan Institute, University of Galway, Galway H91 TK33, Ireland; State Key Laboratory of Advanced Environmental Technology, Institute of Urban Environment, Chinese Academy of Sciences, Xiamen 361021, China; State Key Laboratory of Advanced Environmental Technology, Institute of Urban Environment, Chinese Academy of Sciences, Xiamen 361021, China; State Key Laboratory of Loess Science, Institute of Earth Environment, Chinese Academy of Sciences, Xi’an 710061, China; State Key Laboratory of Loess Science, Institute of Earth Environment, Chinese Academy of Sciences, Xi’an 710061, China; State Key Laboratory of Loess Science, Institute of Earth Environment, Chinese Academy of Sciences, Xi’an 710061, China; State Environmental Protection Key Laboratory of Formation and Prevention of Urban Air Pollution Complex, Shanghai Academy of Environmental Sciences, Shanghai 200233, China; State Key Laboratory of Regional Environment and Sustainability, College of Environmental Sciences and Engineering, Peking University, Beijing 100871, China; School of Natural Sciences, Centre for Climate & Air Pollution Studies, Ryan Institute, University of Galway, Galway H91 TK33, Ireland; State Key Laboratory of Loess Science, Institute of Earth Environment, Chinese Academy of Sciences, Xi’an 710061, China; School of Natural Sciences, Centre for Climate & Air Pollution Studies, Ryan Institute, University of Galway, Galway H91 TK33, Ireland; School of Natural Sciences, Centre for Climate & Air Pollution Studies, Ryan Institute, University of Galway, Galway H91 TK33, Ireland

**Keywords:** marine primary organic aerosols, cloud condensation nuclei, aerosol indirect effect, marine aerosols

## Abstract

Marine primary organic aerosols (mPOAs) play a key role in ocean–atmosphere–climate interactions. Yet their effects on cloud formation remain uncertain because mPOA number size distribution measurements are lacking, obscuring its parameterizations for climate models. The mPOA number size distribution is resolved by combining long-term hygroscopicity, size, number and composition measurements in pristine marine air. The resulting bimodal distribution shows strong Aitken (∼60 nm) and accumulation (∼150 nm) modes, unlike the parameterization in widely used models. Total mPOA number is probably underestimated by a factor of three (observed/parameterized ≈ 3.1), doubling mPOA-originated cloud condensation nuclei across realistic marine supersaturations. An observation-guided sensitivity analysis indicates a potential first aerosol indirect effect of –0.03 to –0.04 W/m^2^ over the global ocean, reaching –0.1 to –0.2 W/m^2^ over biologically active waters when mPOA surface tension reduction is considered, underscoring the need for trustworthy mPOA representation to reduce the uncertainties in aerosol–cloud interactions.

## Teaser

Marine organisms release organic particles that brighten clouds over the ocean. By measuring these particles across the full range of sizes for the first time, we find about three times as many of them as climate models assume, pointing to a larger natural cooling effect over the most biologically productive ocean waters.

## INTRODUCTION

Marine primary organic aerosols (mPOAs) co-emitted with sea spray aerosols, formed by bubble bursting in biologically rich waters [1,2], represent a critical yet poorly constrained component of Earth’s climate system. With global production estimates ranging from 2.8 to 76 Tg per year [3,4], mPOA represents a key pathway for ocean carbon cycling [5,6]. Moreover, field studies have confirmed that mPOA is an important component of marine aerosols [2,7,8]. Due to the generally low background concentration of overall marine cloud condensation nuclei (CCN), the addition of mPOA can strongly influence local cloud brightness [9]. The mPOA also modifies the microphysical properties of sea spray aerosols [10,11]. Therefore, a better understanding of mPOA is crucial for improving future climate projections [12,13].

Despite a significant advancement in understanding of mPOA sources, chemical composition and cloud activation [11,14,15], the knowledge gap is still substantial, which prevents mPOA inclusion into climate models, including those used in the Sixth Assessment Report of the Intergovernmental Panel on Climate Change [13]. Filter-based measurements have provided valuable information on chemical speciation at different sizes, but suffer from poor temporal and size resolution [2,8,16,17]. Most previous studies have thus focused on the mass concentration of mPOA, and models have parameterized mPOA emissions using the mPOA/sea spray mass ratio [17–19]. So far, little is known about the particle number size distribution (PNSD_mPOA_) due to the difficulties of distinguishing mPOA from other aerosol species in ultrafine size ranges (<100 nm) that contribute most significantly to CCN. The lack of PNSD_mPOA_ information from ambient marine air has hindered the understanding of the impact of mPOA on marine CCN populations and aerosol indirect effects (AIEs) [4,20–22].

In this study, we derive PNSD_mPOA_ and *N*_mPOA_ in Northeast Atlantic air masses based on a suite of long-term (5 years) aerosol microphysics and chemical observations with a high-resolution time-of-flight aerosol mass spectrometer (HR-ToF-AMS), a scanning mobility particle sizer (SMPS) and a humidified tandem differential mobility analyzer (HTDMA) as key instruments for mPOA identification. For ease of discussion, the mPOA mass concentration refers to the mPOA bulk concentration measured by AMS and PNSD_mPOA_ refers to the particle number size distribution of mPOA derived from HTDMA. This study represents the first synergistic derivation of PNSD_mPOA_ from combined HTDMA–SMPS–AMS measurements. The derived PNSD_mPOA_ is then used to evaluate the mPOA contribution to marine CCN and first AIEs through an observation-guided sensitivity analysis.

## RESULTS

### Distinct microphysical properties of mPOA

This work is based on long-term aerosol microphysics and chemistry measurements at the Mace Head Atmospheric Research Station, located on the west coast of Ireland, which is frequently exposed to clean marine air masses from the Northeast Atlantic. Rigorous data filtering with black carbon and wind directions ensured the representativeness of clean marine air (see materials and methods) and minimized anthropogenic influences. The chemical composition of bulk PM_1_, including mPOA and marine secondary organic aerosol (mSOA), was probed using an HR-ToF-AMS and was derived in Chen *et al.* [23], using positive matrix factorization (PMF) and machine learning approaches (see materials and methods). To verify the marine origin of mPOA, we calculated Pearson’s correlation coefficients between the bulk PM_1_ concentrations of mPOA, mSOA and other chemical species representative of different sources. As shown in Fig. S1, the mPOA bulk concentration showed no detectable correlation with anthropogenic tracers (NO_3_^−^ and black carbon) or marine secondary species (non-sea-salt sulfate, nss-SO_4_^2−^ and methanesulfonic acid), supporting their primary marine origin.

Strong seasonal variation in mPOA was observed at the Mace Head Atmospheric Research Station, with the highest median concentration recorded in July (Fig. 1a). The concentration is slightly lower than the filter-based values reported previously [17], probably due to the differences in sampling sizes and more conservative criteria for clean marine data selection applied in this study (see materials and methods). Although the median mPOA concentration was relatively low (<0.1 μg m^−3^), peak concentrations frequently reached 0.5 μg m^−3^ and the North Atlantic Ocean was the dominant source region for mPOA (Fig. 1b).

**Figure 1.**
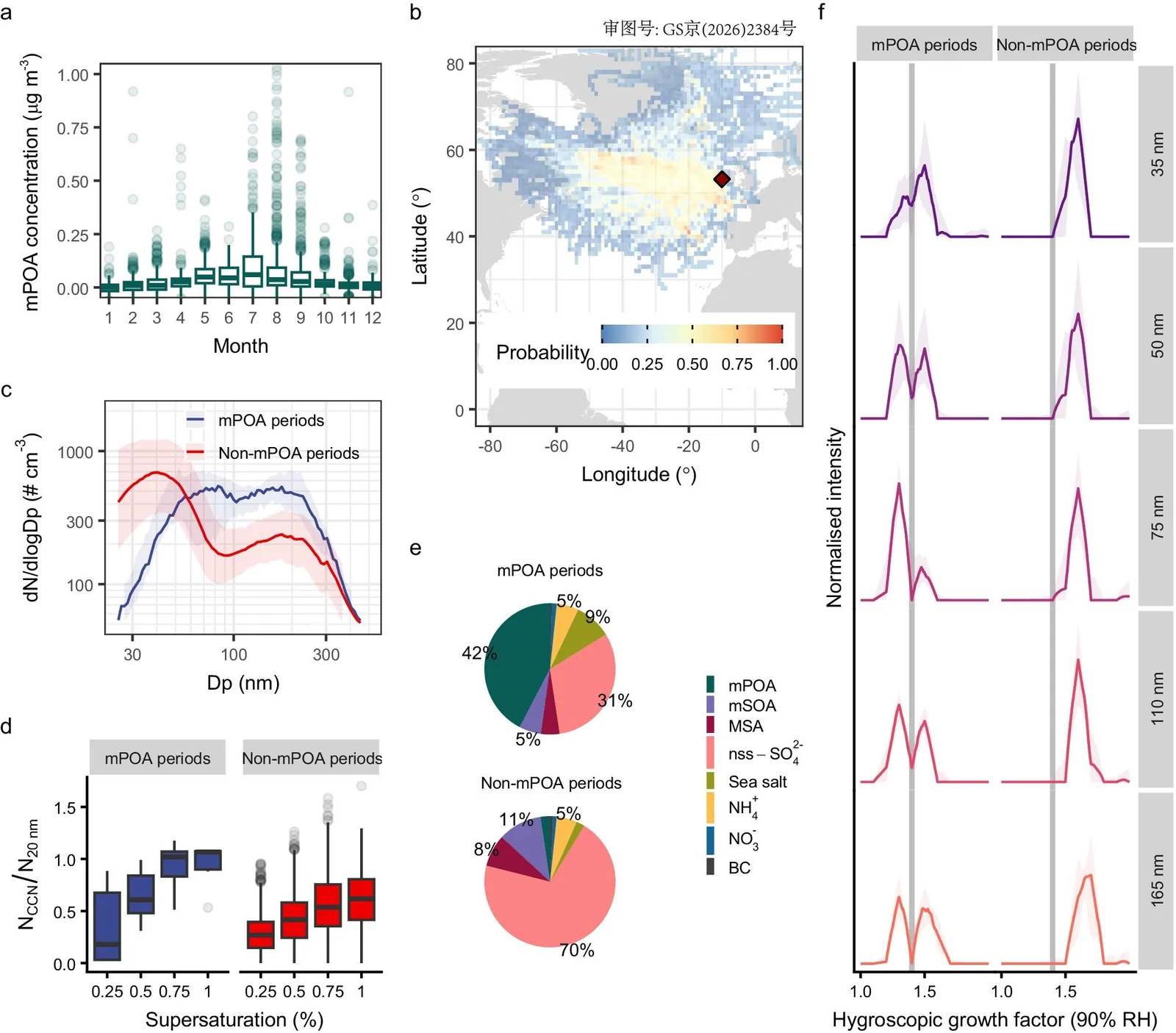
Seasonal variation, source regions and physicochemical properties of mPOAs). (a) Monthly variation in mPOA concentration. (b) Potential source region function analysis of mPOA observed in Mace Head: reddish color scale represents higher probability and brown marker represents the location of Mace Head. (c) Dry particle number size distributions. (d) *N*_CCN_/*N*_20nm_ under different supersaturations (SS). In (a) and (c), horizontal lines represent median values, shaded areas and boxes represent 25th to 75th percentiles and whiskers represent 1.5 interquartile ranges. (e) Percentage contribution of chemical species to PM_1_ and (f) aerosol hygroscopic GF-PDF at 90% RH during mPOA periods (mPOA > 0.5 μg m⁻³) and non-mPOA periods (clean marine, mPOA < 0.5 μg m⁻³). Vertical gray lines represent a GF of 1.4. In (d)–(f), the data are only shown for the warmer seasons (April–August) to minimize the impact of seasonal variations.

We define mPOA periods as clean marine periods with mPOA concentrations exceeding 0.5 μg m^−3^, which is ∼2 s.d. above the mean value of mPOA during the whole period. Most of the mPOA periods were observed during summertime. During the mPOA periods, the mPOA contributed to ∼90% of the total organic aerosol (OA). Significant differences in the dry PNSD were identified between the mPOA and non-mPOA periods (Fig. 1c). During non-mPOA periods (clean marine, mPOA < 0.5 μg m^−3^), PNSD exhibited a typical bimodal marine signature with higher particle number in the Aitken mode [24], whereas mPOA periods showed a stronger contribution in the accumulation mode (100–200 nm). The higher CCN activation ratio (*N*_ccn_/*N*_20nm_) during mPOA periods compared to non-mPOA periods across all supersaturation levels (Fig. 1d) suggests that the mPOA acts as efficient CCN, despite its relatively low hygroscopicity. As the mPOA periods were mostly found during warmer seasons (April–August), only summertime non-mPOA periods were used for comparison. CCN closure analysis indicates that a surface tension reduction of at least 30% is required to reconcile the observed CCN activation with standard κ-Köhler theory under pure-water surface tension (Fig. S12 and related discussion).

Although strong mPOA contributions to total mass were identified, secondary species such as SOA and nss-SO_4_^2−^ were ubiquitous (Fig. 1e), accounting for up to 49% of aerosol mass during the defined mPOA periods, posing challenges for extracting *N*_mPOA_ and PNSD_mPOA_ to guide model development. During mPOA periods, the sea salt contribution was also higher than that during non-mPOA periods, supporting the primary origin of mPOA. The more-hygroscopic mode dominated for non-mPOA periods (Fig. 1f). The bimodal separation between less- and more-hygroscopic modes is statistically significant at all five measured sizes (35–165 nm; Kolmogorov–Smirnov test vs fitted unimodal normal distribution, *P* < 0.01; Hartigan Dip test for multimodality, *P* < 0.01).

This less-hygroscopic mode was attributed to mPOA and used to quantify size-resolved mPOA for the following reasons: The less-hygroscopic mode is a typical mPOA characteristic [10,25] while the more-hygroscopic mode is typical for marine secondary species such as nss-SO_4_^2−^ [26–28]. The bimodal hygroscopic growth factor probability density function (GF-PDF) shown in Fig. 1f indicates that the less-hygroscopic mode—attributed here to mPOA—is externally mixed from the more-hygroscopic mode, which represents internally mixed SOA and nss-SO_4_^2−^. While the mixing state of mPOA with sea salt remains debated, during the mPOA-dominated periods selected for PNSD derivation, Zdanovski–Stokes–Robinson calculations indicate that inorganic sea salt contributes <30% to the less-hygroscopic mode (Fig. S2) with even lower contribution for smaller particles due to increasing organic enrichment with decreasing size [2], supporting the robustness of the hygroscopicity-based separation. We acknowledge that the evidence for the GF < 1.4 classification, while convergent, is indirect. Direct size-resolved chemical speciation capable of probing <100 nm—for instance, via single-particle mass spectrometry or thermal desorption chemical ionization—would provide definitive validation and remains an important direction for future work. The present study nonetheless provides the first size-resolved mPOA number constraints in this size range.

### Bimodal PNSD of mPOA

Figure 2a displays the size-resolved number fraction of mPOA (nf_mPOA_) derived from HTDMA measurements during the mPOA periods, revealing the largest contribution of mPOA to the total number of marine aerosols at 75 nm. Comparison with previous field campaigns highlights substantial variability in the reported mPOA contribution to sea spray aerosols. For instance, Facchini *et al*. [29] observed an mPOA contribution of up to 60% at 200 nm during summertime over Mace Head, while Lawler *et al*. [30] reported a minimal contribution of mPOA during a global flight campaign and O’Dowd *et al*.[2] found that mPOA dominates at smaller sizes (∼90 nm in their filter-based measurement) in the Northeast Atlantic. These discrepancies likely arise from differences in sampling methodologies, analytical techniques and regional oceanic conditions. The size and temporal resolution of HTDMA enable precise derivation of mPOA size distributions, a challenging task for conventional filter-based methods. The mPOA fractions for 110 and 165 nm sizes from HTDMA agree well with the bulk PM_1_ mPOA mass fraction from AMS measurements (Fig. 2b), reconfirming the feasibility of attributing this less-hygroscopic mode to mPOA.

**Figure 2.**
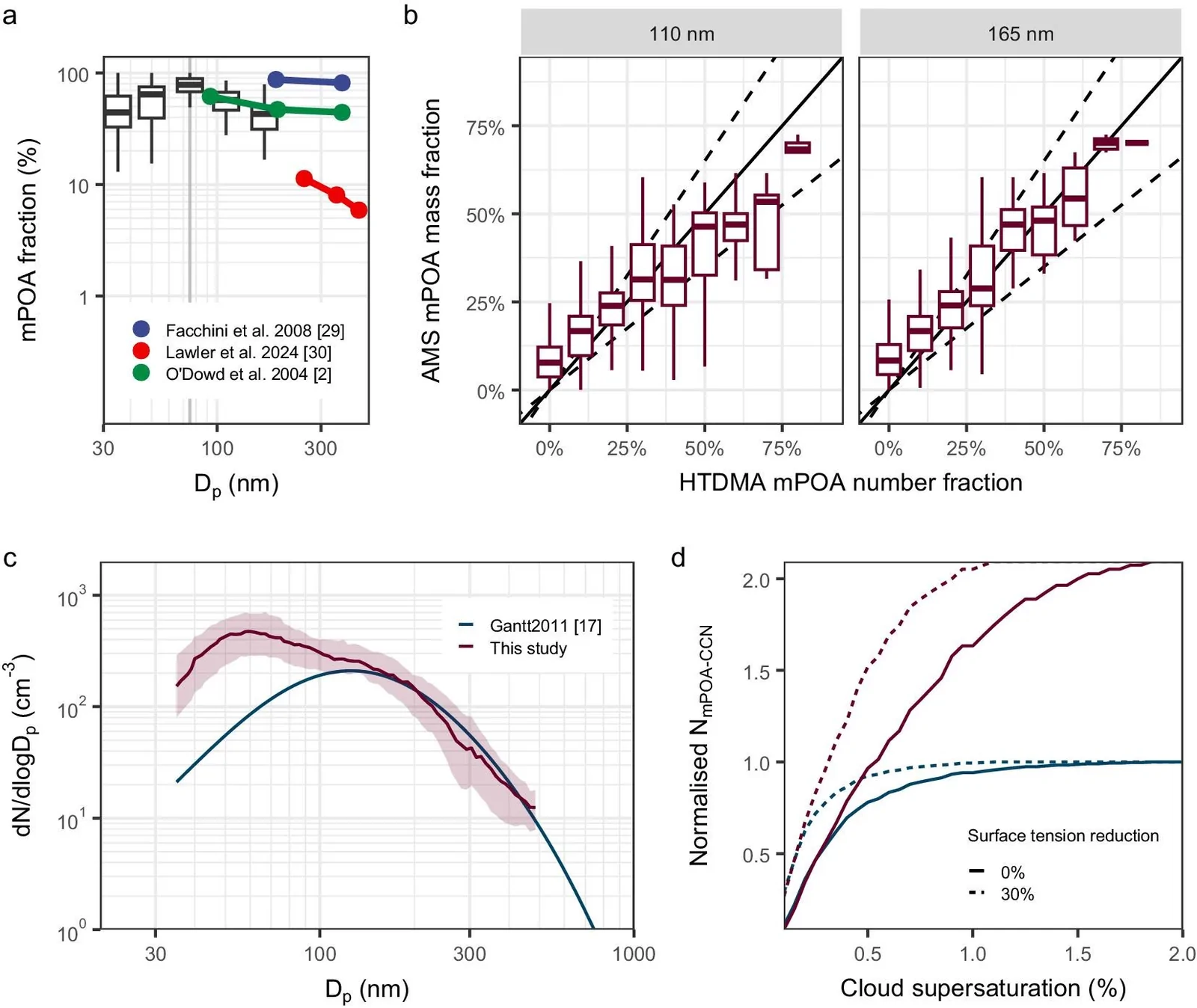
PNSD and CCN contribution of mPOA. (a) Size-resolved fraction of mPOA (nf_mPOA_) to total number obtained by HTDMA. Data from previous studies are shown for comparison. (b) Comparison between size-resolved nf_mPOA_ (obtained by HTDMA) versus mPOA mass fraction (obtained by AMS). In (a) and (b), horizontal lines represent median values, boxes and shaded areas represent the 25th to 75th percentiles and whiskers represent 1.5 interquartile ranges. (c) Calculated PNSD of mPOA from this study (red lines) and from Gantt2011 parameterization (blue line). For the ease of comparison, two PNSD_mPOA_ were merged by minimizing the number weighted residuals for particles >150 nm. (d) Normalized mPOA-originated CCN (*N*_mPOA-CCN_) obtained with this study (red line) and Gantt 2011 (blue line) assuming 0% and 30% surface tension reduction, normalized by *N*_mPOA-CCN_ from Gantt2011 parameterization at supersaturation of 1%.

As shown in Fig. 2c, the PNSD_MPOA_ derived by HTDMA is characterized by two modes, with one centered at 60–70 nm and a broader distribution centered between 100 and 200 nm. While the nf_mPOA_ of 165 nm (the HTDMA upper size limit) is assumed for particles >165 nm for extrapolation, this introduces minor uncertainty in *N*_mPOA_ and PNSD_mPOA_ quantification, as particles in the 35–165 nm size range account for >80% of the total *N*_mPOA_ (Fig. S4). Despite deviations from SMPS–AMS size cut-off differences (SMPS: 500 nm electrical mobility size; AMS: 1 μm vacuum aerodynamic size), the method validation with AMS mass shows a strong agreement (*R* = 0.87) between HTDMA-derived and AMS-measured mPOA volume (Fig. S5). The extracted PNSD_mPOA_ is in stark contrast with polluted air mass distributions at Mace Head (Fig. S6), reinforcing its marine biogenic origin.

The PNSD_mPOA_ from the Gantt2011 mPOA parameterization [17], which is adopted by several widely used models—the Goddard Earth Observing System chemistry model (GEOS-Chem), the UK Earth System Model (UKESM) and the Community Multiscale Air Quality model (CMAQ)—shows a monomodal shape at submicron sizes peaking at 120 nm (Fig. 2c, blue line), with an additional mode in supermicron size (∼2 μm). Gantt2011 mPOA parameterization is a wind speed-dependent scheme developed by combining the organic mass/sea spray ratio [17] and sea spray source flux function from Gong [31]. The mPOA/sea spray ratio was obtained from filter measurements (0.125–8 μm) conducted by Facchini *et al*. [29] at Mace Head. The organic matter/sea spray ratio for particle sizes <0.125 μm was obtained by extrapolation. The shape of the PNSD_mPOA_ from Gantt2011 parameterization is consistent across different environmental conditions (Fig. S7), enabling a direct comparison.

Compared to the measured PNSD_mPOA_, the Gantt2011 parameterization underestimated the total mPOA number (>35 nm) by approximately threefold (*N*_mPOA_measure_/*N*_mPOA_gantt_ = 3.1). Stronger underestimation was observed at smaller sizes, reaching 5–7 folds for Aitken-mode (30–70 nm) particles. This suggests that parameterizations based on filter-derived organic mass fractions may substantially underestimate the sub-100 nm mPOA number flux. Such substantial underestimation likely reduced the predicted marine CCN availability from mPOA. Using the κ-Köhler model, we quantified the difference in mPOA-originated CCN between GEOS-Chem and our PNSD_mPOA_ (see materials and methods). Figure 2d shows that significant mPOA-originated CCN underestimation occurred at supersaturations >0.25%. Larger discrepancies would occur when considering the potential reduction in surface tension induced by mPOA [10,32]. For example, assuming a 30% reduction in surface tension, 16% and 64% mPOA-CCN underestimations would be expected at 0.25% and 0.5% cloud supersaturation, respectively.

### Potential implications for marine cloud brightness

Many earth system models and chemical transport models omit mPOA emissions due to highly uncertain parameterizations with limited observational datasets for validation (summarized in Table S1). Exceptions include GEOS-Chem, UKESM and CMAQ, which adopt parameterization Gantt2011 [17]. While the Energy Exascale Earth System Model and Community Earth System Model-2 employ OCEANFILM (Organic Compounds from Ecosystems to Aerosol: Natural Films and Interfaces via Langmuir Molecular Surfactants [33]), which lacks a size-dependent mPOA representation. Despite the abovementioned progress in recognizing the importance of mPOA in the global ocean atmosphere, their impact on marine CCN and clouds remains unresolved. To obtain an order-of-magnitude estimate of the climatic effect of overlooked mPOA, we simulated the global distribution of mPOA mass for 2019 using the GEOS-Chem model (see materials and methods and Fig. S8). Using the prescribed PNSD_mPOA_ obtained in this study, we convert the mass of mPOA to number concentration and estimate the annual mean *N*_mPOA_ over the global ocean surface to be 30.6 ± 35.8 cm^−3^ (mean ± s.d.). The regionally heterogeneous surface mPOA distribution was driven by sea surface chlorophyll-a (Chl-a) concentrations and wind speeds (Fig. S8). Higher concentrations of mPOA were simulated in biologically active oceans, for example, the summertime Northeast Atlantic (40–60°N, 20°W–5°E), where the *N*_mPOA_ was ∼144.5 ± 92.4 cm^−3^, accounting for a substantial fraction of marine aerosol numbers. An increase in aerosol number concentrations within the cloud topped boundary layer leads to an increase in the cloud droplet number concentration (*N*_d_), thereby increasing the cloud albedo and cloud brightness.

Using satellite measurements of cloud properties (Fig. S9) and the GEOS-Chem model of CCN burden (materials and methods), we made an order-of-magnitude estimation of the first AIE from the additional Aitken mode mPOA compared to Gantt2011 (default GEOS-Chem setting). As shown in Fig. 3, the additional Aitken mode mPOA induces ∼–0.03 to –0.04 W m^−2^ at cloud supersaturation of 0.25%. Although the AIE is moderate over the globe, it is significant over biologically active waters. For example, the additional Aitken mode mPOA increases AIE by as much as –0.051 and –0.069 W m^−2^ over the June, July and August (JJA) Atlantic and December, January and February (DJF) Southern Oceans, respectively, for a cloud supersaturation of 0.25%, indicating a strong influence on the radiative budget.

**Figure 3.**
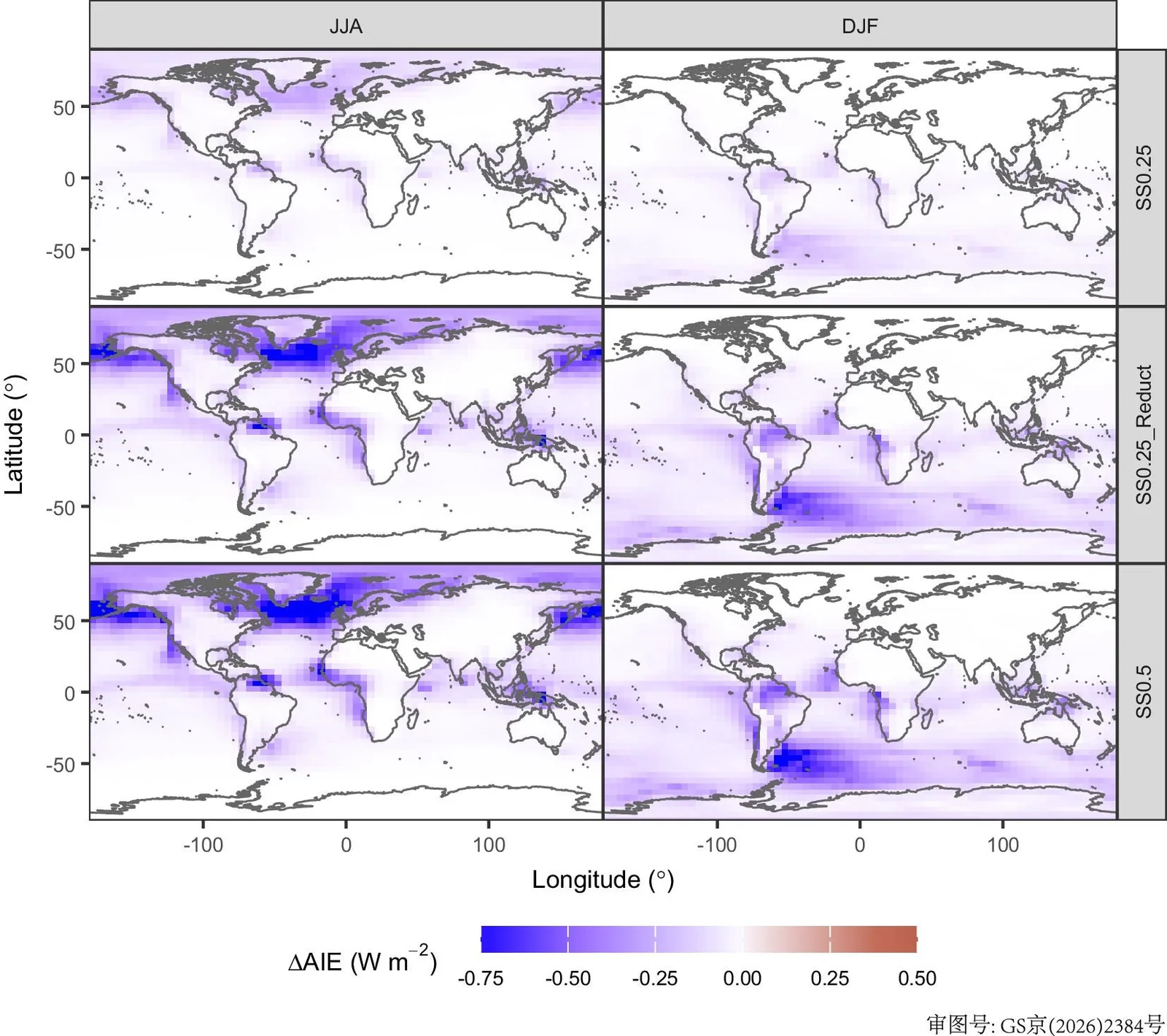
Global distribution of the difference in the first ΔAIE induced by the Aitken mode mPOA compared to the Gantt2011 parameterization. SS0.25_Reduct represents the assuming of surface tension reduction by 30% as a sensitivity calculation, and JJA and DJF represent seasons. Negative values (blue shading) indicate a radiative cooling effect due to increased cloud albedo.

As a sensitivity calculation, the surface tension reduction is used here as an effective parameter to compensate for the deviation from κ-Köhler theory under pure-water surface tension (Fig. 1c); it does not capture surfactant partitioning between the bulk and surface phases or droplet-size-dependent surface tension (Kelvin effect), and ignoring these processes may lead to an overestimate of CCN activity. Frossard *et al.* [34] demonstrated that the surface tension of mPOA was as low as 30 mN m^−1^ (∼60% reduction compared to that of the air–pure water interface [72 mN m^−1^]). Similarly, Kleinheins *et al.* [35] found that a high content of organic matter in the Aitken mode does not necessarily prevent CCN activation but rather facilitates effective activation via surface tension lowering. Therefore, a moderate and conservative estimate of 30% surface tension reduction was assumed, which induced global mPOA AIE up to –0.072 to –0.11 W m^−2^ at cloud supersaturation of 0.25%. Stronger AIE enhancement can be found when considering surface tension reduction over biologically productive waters, such as –0.190 and –0.138 W m^−2^ over the DJF Southern Ocean and JJA North Atlantic. Stronger AIEs are expected for clouds with higher supersaturations [36]. A systematic uncertainty analysis spanning 108 parameter combinations of particle density (1.0, 1.4 and 1.6 g cm^−3^), hygroscopicity κ (0.05, 0.10 and 0.20), cloud supersaturation (0.10%, 0.20%, 0.25% and 0.50%) and surface-tension ratio *σ*/*σ*_w_ (1.00, 0.85 and 0.70) shows that the diagnosed ΔAIE is strongly parameter-dependent (Fig. S13). The central case (*ρ* = 1.4 g cm^−3^, κ = 0.10, SS = 0.25%, *σ*/*σ*_w_ = 1.00) yields global-mean values of ∼−0.03 W m^−2^ in DJF and −0.04 W m^−2^ in JJA, while larger values approaching −0.1 W m^−2^ occur only under the most sensitive parameter combinations, particularly higher supersaturation coupled with lower surface tension. Thus, the calculated ΔAIE should be interpreted as an order-of-magnitude sensitivity estimate rather than a comprehensive climate forcing. A more complete assessment of its climatic importance will require cloud-resolving or climate-model simulations that explicitly represent cloud adjustments in liquid water path, cloud fraction, precipitation and cloud organization. This simplified framework does not account for cloud lifetime effects (the Albrecht effect), precipitation suppression, liquid water path (LWP) adjustments. Comprehensive modelling studies are needed to provide robust quantification of the climatic impact of mPOA.

## DISCUSSION

The mPOA significantly influences marine aerosol–cloud interactions by constituting a critical component of marine aerosol emissions and by modifying aerosol hygroscopicity [10]. However, most previous works on mPOA have focused on its mass concentration rather than number concentration, which determines its contribution to marine cloud formation. This omission limits our ability to fully understand the aerosol and CCN budget within the marine boundary layer. Recently, a growing body of research has begun incorporating mPOA into models [20,33,37,38], highlighting its critical role in marine cloud brightening [39] and ocean carbon cycling [6]. Nevertheless, major challenges persist. Accurately modelling the climate effect of mPOA requires detailed knowledge of its microphysical properties, particularly its size distribution. The mPOA size distribution holds key information about its potential to activate as cloud droplets but is currently lacking due to the measurement difficulties in distinguishing mPOA from other aerosol species, such as nss-SO_4_^2−^ in ambient marine air.

Utilizing the unique hygroscopicity signature of mPOA and comprehensive aerosol microphysics measurements at the Mace Head Atmospheric Observation Station, we derived the PNSD of mPOA down to 35 nm size. Building on this observational constraint, we found a substantial contribution of Aitken mode mPOA to the total mPOA number population. The Gantt2011 parameterization underestimates *N*_mPOA_ by approximately a factor of 3, so models adopting this scheme are likely to underestimate the mPOA contribution to natural cloud brightening. Whether other mPOA schemes (e.g. OCEANFILM) exhibit similar biases in the Aitken mode remains to be investigated. Our PNSD thus provides a constraint relevant to all models that seek to represent the CCN activity of mPOA. Understanding how PNSD_mPOA_ varies with environmental factors—such as biological activity, phytoplankton community composition, wind speed and sea state—is critical for developing trustworthy mPOA source functions. We emphasize that the global first AIE estimates presented here are best viewed as a sensitivity analysis that quantifies the potential magnitude of mPOA effects if the Mace Head-derived PNSD_mPOA_ proves to be representative of global oceans. The Chl-a concentrations and wind speeds at Mace Head span ∼0.1–10 mg m^−3^ and 5–20 m s^−1^, respectively, covering a substantial fraction of the global ocean parameter space, but regional variations in phytoplankton community composition, sea state and atmospheric processing may alter the PNSD_mPOA_. As the measurements were conducted at a single site, extrapolating the current PNSD_mPOA_ findings should be done with great caution. Future efforts should prioritize size-resolved mPOA identification across global oceans, particularly in regions with strong biological activity, such as the Southern Ocean, to test the representativeness of the PNSD_mPOA_ derived here.

Our findings have implications for the representation of natural marine aerosol sources in climate models and for natural marine clouds. Due to the low background of aerosol availability and the strong light-absorbing capability of the underlying dark ocean surface, the radiative properties of marine clouds are extremely sensitive to aerosol perturbations. A modelling study has suggested that even small changes in marine aerosol background (0–40 cm^−3^) can offset 80% of anthropogenic aerosol forcing [40]. Marine phytoplankton are important sources of marine aerosols and play essential roles in natural marine cloud brightening [41,42]. Therefore, over biologically productive ocean regions, the mPOA contribution to cloud radiative properties is likely underestimated in parameterizations that lack an Aitken mode, such as Gantt2011. Previous studies have suggested the strong surface tension reduction of mPOA when interacting with water vapor [7,32]. Accounting for the surface tension reduction further exacerbates its contribution to marine CCN. Furthermore, high CCN concentrations also inhibit the formation of drizzles, resulting in a higher liquid water content and longer cloud lifetime, further increasing its cooling effect. The substantial contribution of Aitken mode mPOA to the natural CCN reservoirs has implications for assessing the potential efficacy of marine cloud brightening geoengineering strategies, as the mPOA defines a significant preexisting CCN background [43]. As oceans warm, enhanced water vapor supply will likely increase cloud supersaturation, amplifying the role of smaller CCN, such as Aitken mode mPOA, in natural marine cloud brightening.

## MATERIALS AND METHODS

### Site and measurements

Measurements were conducted from 2009 to 2015 at the Mace Head Atmospheric Research Station (53°19′N, 9°54′W), on the west coast of Ireland, which is frequently exposed to clean marine air masses from the Northeast Atlantic. Aerosol microphysical, hygroscopic, CCN and chemical composition measurements were made with an SMPS, an HTDMA operating at 90% relative humidity (RH), a streamwise CCN counter, an HR-ToF-AMS and a multi-angle absorption photometer; instrument specifications, operating parameters and quality assurance are given in the Methods S1.

### Derivation of the mPOA number size distribution

The mPOA mass concentration was obtained by combining machine learning with PMF of the high-resolution organic mass spectra [23] (Methods S2). mPOA-dominated periods (mPOA > 0.5 μg m^−3^, ∼2 standard deviations above the mean) were selected; during these periods mPOA accounted for ∼90% of the OA. Within these periods, particles with GF < 1.4 at 90% RH were classified as mPOA, the threshold being chosen from the bimodal GF-PDF. PNSD_mPOA_ was obtained by integrating the GF-PDF below GF = 1.4 at each dry diameter and weighting by the total SMPS number size distribution (Methods S3); the 165 nm fraction was assumed for larger particles.

### CCN number calculation and uncertainty quantification

The mPOA-originated CCN number concentration was obtained by summing the particles above the critical activation diameter (*D*_crit_) from κ-Köhler theory with a hygroscopicity parameter κ = 0.1 [44]. CCN were evaluated at supersaturations of 0.25% and 0.5%, a range representative of marine liquid clouds [45–47] (Methods S4). Primary results use pure-water surface tension. A conservative 30% surface tension reduction was evaluated as a sensitivity case [10,32], deliberately lower than the 34% median reduction required to close the observed CCN budget at 0.5% supersaturation (Fig. S12) so as to avoid overestimating CCN activity. A Monte Carlo propagation (*N* = 1000) quantified uncertainty from the GF threshold, AMS collection efficiency, SMPS counting statistics and OA hygroscopicity (Methods S5; Tables S2 and S3) and the mPOA event threshold, >165 nm extrapolation scheme and the black carbon (BC) screening criterion were each tested for sensitivity.

### Data screening

Clean marine sectors were selected using a black carbon criterion < 15 ng m^−3^ and a wind direction between 190° and 300° [48].

### GEOS-Chem simulation

Global simulations were conducted with the GEOS-Chem chemical transport model (v14.6.2) for 2019, using the Gantt2011 wind-speed-dependent mPOA emission scheme [3,17], in which the mPOA mass fraction of sea spray depends on satellite-derived Chl-a concentration and 10-m wind speed. The model configuration and the mass-to-number conversion are given in the Methods (S6).

### Aerosol indirect radiative effect calculation

The first aerosol indirect radiative effect was estimated following Meskhidze and Nenes [49]. Assuming a constant liquid water path (Twomey effect) [50], the reflected solar flux is scaled by the relative change in total CCN concentration. ΔAIE was computed from the downwelling shortwave at the cloud top, the moderate resolution imaging spectroradiometer (MODIS) cloud fraction and albedo and the relative change in cloud droplet number (Methods S7). This framework excludes cloud lifetime effects, precipitation suppression and liquid water path adjustments; the resulting ΔAIE is therefore an order-of-magnitude, observation-guided sensitivity estimate rather than a comprehensive radiative forcing assessment.

## Supplementary Material

nwag604_Supplemental_File

## Data Availability

All data are available in the repository: http://doi.org/10.17632/v5j665z649.1. The codes used to analyze the data are available upon request. The GEOS-Chem model used in this study is available at https://geoschem.github.io. CERES data are available at https://ceres.larc.nasa.gov/data/. MODIS data are available at https://www.earthdata.nasa.gov.
