# Supplementary material for: Natural cloud brightening enhanced by marine primary organic aerosols
Source: Natl Sci Rev. 2026 Sep 17;13(20):nwag604. doi: 10.1093/nsr/nwag604 (PMC13702025; doi:10.1093/nsr/nwag604)
Supplement: Supplementary file 1 [file nwag604_supplemental_file.docx]

Supplementary information for ‘Natural cloud brightening enhanced by marine primary organic aerosols’

Wei Xu^1^†, Baihua Chen^1^†, Lin Wang^1^†, Keran Zhang^1^, Lu Lei^2^, Kirsten Nicole Fossum^2^, Emmanuel Chevassus^2^, Liyuan Zhou^1^, Haobin Zhong^1^, Chunshui Lin^3^, Yanan Zhan^3^, Jingye Ren^3^, Dan Dan Huang^4^, Ruqian Miao^5^, Darius Ceburnis^2^, Ru-Jin Huang^3^, Colin O’Dowd^2^, Jurgita Ovadnevaite^2^

^1^State Key Laboratory of Advanced Environmental Technology, Institute of Urban Environment, Chinese Academy of Sciences, Xiamen, China.

^2^School of Natural Sciences, Centre for Climate & Air Pollution Studies, Ryan Institute, University of Galway, University Road, Galway, Ireland.

^3^State Key Laboratory of Loess Science, Institute of Earth Environment, Chinese Academy of Sciences, Xi’an, China.

^4^State Environmental Protection Key Laboratory of Formation and Prevention of Urban Air Pollution Complex, Shanghai Academy of Environmental Sciences, Shanghai, China.

^5^State Key Laboratory of Regional Environment and Sustainability, College of Environmental Sciences and Engineering, Peking University, Beijing, China.

†These authors contributed equally to this work.

# Supplementary Methods

## S1. Instrument specifications and quality assurance

**Aerosol number size distribution.** Aerosol size distributions (20 to 500 nm) were measured using a scanning mobility particle sizer (SMPS), consisting of a differential mobility analyzer (DMA) and a condensable particle counter (CPC), with measurements taken every 10 minutes. The SMPS system followed the particle physical properties measurement protocol set by the European Supersites for Atmospheric Aerosol Research (EUSAAR).

**Sub-saturation hygroscopicity.** Hygroscopic growth factor probability density functions (GF-PDF) were measured using a Hygroscopic Tandem Differential Mobility Analyzer (HTDMA)[1-4]. Details of the HTDMA at Mace Head can be found in previous studies[5,6]. The HTDMA consists of two DMAs, a temperature-controlled humidifier, and a CPC. Monodispersed particles of selected electrical mobility were first chosen by applying a voltage to DMA-1. The particles were then humidified using a Gex-Tex membrane humidifier and scanned by DMA-2, which was operated with varying voltage. By counting the particles at different voltages, GF-PDF was obtained across a size range from 35 to 165 nm. The GF was calculated by measuring the aerosol size distribution at fixed RH. To extract the GF-PDF from raw data and correct for broadening of the DMA distribution, a piecewise linear inversion algorithm was applied[7]. In this study, DMA-1 was operated at RH below 10%, while DMA-2 was set to 90% RH. The electrical mobility diameters selected by DMA-1 were 35, 50, 75, 110, and 165 nm. The main and sheath flow rates were 1 and 9 L min⁻¹, respectively. The operation and quality assurance followed the standard protocols recommended by the Aerosol, Clouds and Trace Gases Research Infrastructure (ACTRICS).

**Chemical composition.** The chemical composition of aerosols, including organic matter (Org), ammonium (NH₄⁺), non-sea-salt sulfate (nss-SO₄²⁻), nitrate (NO₃⁻), and methanesulfonic acid (MSA), was measured using a high-resolution time-of-flight aerosol mass spectrometer (HR-ToF-AMS, Aerodyne Research Inc., Billerica, MA)[8]. The HR-ToF-AMS has a vacuum aerodynamic cut-off diameter of 1 μm. Regular calibrations were performed using ammonium nitrate. The AMS typically operates at an evaporation temperature of 600 °C, optimized for detecting nonrefractory aerosol species such as Org, NO₃⁻, nss-SO₄²⁻, and NH₄⁺. The ^23^Na^35^Cl⁺ ion signal at m/z 58, along with a scaling factor of 51, was used to calculate sea-salt concentration[9]. The ion signals of CH₃SO₂⁺ and CH₃SO₃H⁺, specific to MSA, were used for quantification and calibration of MSA[10]. To correct for composition-dependent collection efficiency, the AMS species concentrations were adjusted accordingly[11]. Further details on HR-ToF-AMS operations can be found in our previous study[10]. A multi-angle absorption photometer (MAAP, Thermo Fisher Scientific model 5012) was used to measure the black carbon (BC) concentration. The MAAP operated at a flow rate of 10 L min⁻¹ and measured the transmittance and reflectance of BC-containing particles at two angles to calculate optical absorbance[12].

**CCN measurements.** Cloud condensation nuclei (CCN) number concentrations were measured using a continuous-flow streamwise thermal-gradient CCN counter (CCN-100, Droplet Measurement Technologies) at supersaturations of 0.1%, 0.25%, 0.35%, 0.5%, 0.75%, and 1.0%. The instrument was calibrated using ammonium sulfate particles following standard procedures. The CCN measurements were matched to the SMPS total particle number concentration (N_20nm_) to calculate the CCN activation ratio (N_CCN_/N_20nm_).

## S2. Machine learning retrieval of the mPOA mass concentration

The concentration of mPOA was obtained by combining machine learning and positive matrix factorization on high-resolution organic mass spectra. Full details of the machine learning model and its validation can be found in our previous work[13]. Briefly, we selected the data by wind speed below 6 m/s, sea salt mass concentration under 0.03 μg m^-3^ and nss-SO_4_^2-^ as the dominant component (nss-SO_4_^2-^/OA > 4) to ensure a predominantly secondary source. We presumed that in these selected data, OA was predominantly SOA, with the contribution of mPOA represented by a minor and constant background concentration (POA_bg_). Secondary tracers, including nss-SO_4_^2-^, MSA, NH_4_^+^, meteorological parameters (temperature, relative humidity, boundary layer heights, wind direction and pressure), and time variables (hour of the day and month of the year), were used as predictors. A machine learning model (support vector regressor) was trained on selected clean marine secondary organic aerosol (SOA) production periods. The hyperparameters, including the penalty coefficient (C) and gamma (γ) of the radial basis functions (RBF) kernel, were tuned via grid research. Finally, the mPOA concentration was obtained by subtracting the predicted SOA from the total OA.

## S3. Derivation of the mPOA number size distribution

The derivation of PNSD_mPOA_ follows a step-by-step methodology: (i) select mPOA-dominated periods (mPOA > 0.5 μg m⁻³, approximately 2σ above the mean), during which mPOA accounts for ~90% of total OA (Fig. 1e); (ii) measure size-resolved GF-PDFs at five HTDMA dry diameters (35, 50, 75, 110, 165 nm); (iii) compute the size-resolved nf_mPOA_ by integrating the GF-PDF below GF = 1.4 at each size; (iv) the nf_mPOA_ within the size range of 0.035 to 0.165 μm was determined through linear interpolation, which was then weighted by particle number to obtain the number size distribution of mPOA in this size range; the mPOA fraction at 165 nm (the HTDMA upper size limit) is assumed for particles >165 nm for extrapolation; (v) multiply by the total SMPS PNSD to obtain PNSD_mPOA_. For each GF-PDF, clean marine aerosols with a GF lower than 1.4 were classified as mPOA. This cutoff of 1.4 was chosen based on the bimodal GF-PDF distribution observed during mPOA-dominated events. The mPOA number fraction was computed as

$$nf_{mPOA}(D_{0})=\int_{0}^{1.4} c(GF,D_{0})dGF$$

where c(GF, D_0_) is the GF-PDF at dry diameter D_0_.

The attribution of the less-hygroscopic mode to mPOA is supported by multiple lines of convergent evidence: mPOA-dominated aerosol at Mace Head has characteristically low hygroscopicity[14]; the bimodal GF-PDF separation is statistically significant at all five sizes (KS test and Hartigan Dip test, both p < 0.01); ZSR calculations constrain sea-salt contribution to <20–30% of the less-hygroscopic mode mass (Fig. S2); and single-particle measurements confirm external mixing of sub-200 nm marine organics with sea salt[15,16].

## S4. CCN number calculation

For a given critical supersaturation (Sc), the contribution of CCN by mPOA was calculated by summing up the number of all particles in the mPOA number size distribution larger than the critical diameter (Dc), which was obtained by κ-Köhler theory[17]. The parameter A in the κ-Köhler equation is

$$A=\frac{4\sigma_{s/a}\nu_{w}}{RT\rho_{w}}$$

where T is the temperature (298.15 K), R is the universal gas constant (8.315 J K^-1^ mol^-1^), ρ_w_ is the density of water (997.1 kg m^-3^), M_w_ is the molar mass of water (0.018015 kg mol^-1^), and σ_s/a_ is the surface tension in the interface of droplet and air (assumed to be that of pure water, 0.072 mN m^-1^). The N_CCN_ contributed by mPOA was calculated by adding the number of mPOA with electrical mobility (D_mob_) larger than D_crit_.

Surfactant partitioning between the bulk and surface phases and droplet-size-dependent surface tension were not accounted for; the surface tension reduction is used here as an effective parameter to compensate for the deviation from κ-Köhler theory under pure-water surface tension.

The critical activation diameter and the mPOA-originated CCN number concentration are then obtained as

$$D_{crit}=\sqrt[3]{\frac{4A^{3}}{27\kappa ln^{2}Sc}}$$

$$N_{CCN-mPOA}=\int_{D_{crit}}^{10 \mu m} \frac{dN}{dlogD_{p}}dlogD$$

## S5. Sensitivity analyses and uncertainty quantification

**Uncertainty quantification.** A Monte Carlo (MC) uncertainty propagation (N = 1000) was implemented to quantify how uncertainties in the GF threshold, AMS collection efficiency (CE), and SMPS counting statistics propagate through the full analysis chain — from nf_mPOA_ to PNSD_mPOA_ to CCN concentration. The perturbed parameters and their uncertainty ranges are summarised in Table S2; the ensemble results are summarised in Table S3.

**mPOA event threshold.** The definition of mPOA events, using an mPOA > 0.5 μg m⁻³ criterion (approximately 2σ above the mean), was tested in a sensitivity analysis using alternative thresholds of 0.2, 0.3, and 1.0 μg m⁻³ (Fig. S10). We assumed constant PNSD_mPOA_ shape with only the magnitude of mPOA varying over time. Although this assumption may introduce some uncertainty, it is consistent with current mPOA modelling approaches. As shown in Fig. S7, the PNSD_mPOA_ from the Gantt2011 parameterization remains consistent across different environmental conditions.

**Extrapolation above 165 nm.** Sensitivity tests with three alternative extrapolation schemes (nf_mPOA_ at 500 nm same as 165 nm, reduced by 0.05, and reduced by 0.10) show total N_mPOA_ differs by <4% and CCN at 0.5% supersaturation differs by <8% across schemes (Fig. S11).

**Data inventory and BC threshold.** To minimize the impact of different species, we carefully selected data dominated by mPOA to derive the PNSD_mPOA_. There are over 1029 hours of HR-ToF-AMS data with mPOA higher than 0.2 µg m⁻³ and 211 hours with mPOA higher than 0.5 µg m⁻³, corresponding to HTDMA-SMPS data of 371 hours and 70 hours. A sensitivity analysis using BC thresholds of 10 and 15 ng m⁻³ confirmed that the derived PNSD_mPOA_ is insensitive to the specific BC criterion (Fig. S14), reflecting the dominant role of the clean sector wind direction filter (190–300°) in separating marine from continental air masses at Mace Head.

## S6. GEOS-Chem model configuration and mass-to-number conversion

We conducted global simulations using the GEOS-Chem (<http://geos-chem.org>, version 14.6.2) chemical transport model to simulate the mass concentration of mPOA. All simulations were conducted with a 4.0°(latitude) × 5.0°(longitude) resolution at 47 vertical levels. Meteorological fields from the Modern-Era Retrospective Analysis for Research and Applications, Version 2 (MERRA-2) were used to drive the simulations. Global anthropogenic emissions were obtained from the Community Emission Data System (CEDS). Version 4 of the Global Fire Data (GFED4) database was used for open biomass-burning emissions[18]. Biogenic volatile organic compound emissions were calculated by the Model of Emissions of Gases and Aerosols from Nature version 2.1 (MEGAN 2.1[19]). Terrestrial organic aerosol emissions including bio fuel and fossil fuel were based on ref.[20]. The model was run with a full-chemistry configuration, which includes H_2_SO_4_-HNO_3_-NH_3_ aerosol thermodynamics (ISORROPIA II) coupled to an O_3_-NO_x_-hydrocarbon aerosol chemical mechanism[21,22]. A year-long GEOS-Chem simulation for 2019 was performed for the global domain, with a 6-month spin-up. Submicron mPOA emission schemes were used[23,24] as follows:

$$mPOA_{SSA}(chl a,U10,Dp)=\frac{(\frac{1}{1+exp(-2.63[chl a])+0.18(U_{10})})}{1+0.03exp(6.81D_{p})}+\frac{0.03}{1+exp(-2.63[chl a]+0.018(U_{10})}$$

where mPOA_SSA_ represents the mPOA mass fraction of SSA, the emission rates are exclusively related to chlorophyll a concentration ([chl a]) and linked to size-dependent SSA emission, U10 represents wind speed at 10 meters height, and Dp is the diameter of SSA. The daily average chlorophyll-a concentration (<https://oceancolor.gsfc.nasa.gov>) was used in this study to calculate mPOA emissions. SSA emissions in the model were classified into two size bins: fine mode (ranging from 0.02 to 1.0 µm in diameter) and coarse mode (ranging from 1.0 to 16.0 µm in diameter), both of which were dependent on 10-m wind speeds[25.] for both fine and coarse-mode SSA. A detail description of mPOA simulation can be found in Ref[24].

Since the GEOS-Chem output provides only bulk mass concentrations, we utilized the parameterization[26,27] to convert the mass concentration (M) to number concentration (N). Assuming log-normal distributions of each aerosol, the total number concentration was derived directly from the mass concentration as:

$$N=\frac{M}{\rho}(\frac{\pi}{6}D_{p}^{3}exp(\frac{9}{2}ln^{2}\rho_{p}))$$

where ρ represents the particle density and *D_p_* and *σ_p_* represent the geometric mean diameter and the standard deviation of the number size distribution, respectively. Consistent with the GEOS-Chem model configuration[25.] and previous studies[28], we adopted the bimodal lognormal size distribution in sea salt calculation (D_p_ = 0.18 µm and σ = 1.5 for the accumulation mode and D_p_ = 0.80 µm and σ = 1.8 for the coarse mode), with a density of 2.17 g/cm^3^. Sulfate aerosols were represented by a single lognormal mode (D_p_ = 0.14 µm and σ = 1.6) with a density of 1.85 g/cm^3^. The number concentrations for sea salt and non-sea-salt sulfate aerosols were subsequently used for CCN activation analysis, with hygroscopicity parameters of 0.6 and 1.1, respectively[17]. The mPOA, SSA and non-sea-salt sulfate were treated externally mixed as in the GEOS-chem default setting[23]. The marine secondary organic aerosols was not included in the simulation due to the large uncertainty in emission inventories of marine volatile organic compounds. A density sensitivity analysis spanning ρ = 1.2–1.4 g cm⁻³ for mPOA is provided in Table S4. Varying ρ from 1.2 to 1.4 g cm⁻³ changes the mass-to-number conversion factor by approximately 17%. Notably, the CCN enhancement ratio relative to the Gantt2011 parameterization is insensitive to the assumed density, because the same density enters both calculations.

## S7. Aerosol indirect effect calculation and data sources

The first aerosol indirect radiative effect was estimated following Meskhidze & Nenes[29]. Assuming a constant liquid water path (Twomey effect)[30], the reflected solar flux is scaled by the relative change in total CCN concentration:

$$\Delta AIE=-\frac{1}{3}F_{in}A_{c}R_{c}(1-R_{c})\Delta lnNdb$$

$$\Delta lnNdb=\frac{N_{enhanced}-N_{gantt}}{N_{total}}$$

where F_in_ is the downwelling shortwave at the cloud top, A_c_ the monthly MODIS cloud fraction, R_c_ the monthly MODIS cloud albedo, N_enhanced_ − N_gantt_ the net increase in activated droplets due to Aitken mode mPOA relative to Gantt2011, and N_total_ the total cloud droplet number.

Downwelling SW at the cloud top was obtained from the Clouds and Earth’s Radiant Energy Systems (CERES) Energy Balanced and Filled (EBAF) 2.8 downwelling clear-sky SW at the surface[31]. Cloud albedo was estimated from the MODIS cloud optical depth[32]. Both cloud fraction and cloud optical depth were collected from the MODIS Collection 6 MYD08_M3 dataset[33].

Table S1. The mPOA schemes used in earth system models or chemical transport models.

| model | sea-salt emission | mPOA emission | reference |
| --- | --- | --- | --- |
| UKESM | GO03 | Gantt2011 | [34] |
| CESM2 | MO86, MA03 | OCEANFILM | [35,36] |
| E3SMv2 | MO86 | OCEANFILM | [37-39] |
| GEOS-Chem | JA11 | Gantt2011/2015 | [24] |
| CMAQ | JA11 | Gantt2011 | [40] |
| NorESM2 | SA15 | Vignati2010 | [41,42] |
| BCC-ESM1 | MO86 | NA | [43] |
| CNRM-ESM | JA11, GR14 | NA | [44] |
| EC-Earth | GO03 | NA | [45,46] |
| GISS-E2.1 | MO86 | NA | [47,48] |
| IPSL-CM6A | MO86, SM98 | NA | [49,50] |
| MICRO-ES2L | MO86 | NA | [51] |
| MPI-ESM | MO86, SM98 | NA | [52,53] |
| MRI-ESM | MO86 | NA | [54] |
| CanESM | LS04 | NA | [55,56] |
| GFDL-ESM3 | JA11 | NA | [57] |

Full model names:

The Community Earth System Model — CESM2

The Centre National de Recherches Météorologiques Earth System Model — CNRM-ESM2-1

The European Community Earth System Model — EC-Earth3-AerChem

The NASA Goddard Institute for Space Studies Earth System Model — GISS-E2-1-H

The Hadley Centre Global Environmental Model — HadGEM3-GC31-LL

The Institut Pierre Simon Laplace Climate Model — IPSL-CM6A-LR-INCA

The Model for Interdisciplinary Research on Climate Earth System for Long-term Simulations — MIROC-ES2L

The Max Planck Institute Earth System Model — MPI-ESM-1-2-HAM

The Meteorological Research Institute Earth System Model — MRI-ESM2-0

The Norwegian Earth System Model — NorESM2-LM

The UK Earth System Model —UKESM1-0-LL

The Canadian Earth System Model version 5—CanSEM

The Community Multiscale Air Quality Model—CMAQ

The Goddard Earth Observing System-Chem model—GEOS-Chem

MA06 is Mahowald et al. (2006)[58], MO86 is Monahan et al. (1986)[59], MA03 is Mårtensson et al. (2003)[60], JA11 is Jaeglé et al. (2011)[25.], GR14 is Grythe et al. (2014)[61], GO03 is Gong (2003)[62], SA15 is Salter et al. (2015)[63], and SM98 is M. H. Smith and Harrison (1998)[64]. LS04 is Lewis and Schwartz (2004)[65]. Gantt2011 is Gantt et al. (2011)[23], OCEANFILM is Burrow et al. (2014)[66] and Vignati2010 is Vignati et al. (2010)[67].

To note that JA11 is derived from GO03 with additional incorporation of sea surface temperature. The shape of size distribution from JA11 is similar to GO03.

All models listed above include dimethyl sulfide (DMS) oxidation chemistry (DMS → MSA + SO₂ → sulfate). Methanesulfonic acid (MSA, CH₃SO₃H) is an organic sulfur compound tracked within the sulfur budget, not a carbonaceous secondary organic aerosol (SOA). None of the 16 models includes an explicit formation pathway for carbonaceous marine secondary organic aerosol (mSOA) from ocean-emitted volatile organic compounds such as marine isoprene or monoterpenes. Of the 16 models surveyed, 6 include an explicit mPOA scheme; the remaining 10 models treat neither primary nor secondary marine organic aerosol.

Table S2. Parameters perturbed in the Monte Carlo uncertainty propagation.

| Parameter | Distribution | Range / Description |
| --- | --- | --- |
| GF threshold for mPOA identification | Uniform | 1.30–1.50 (±7%) |
| AMS collection efficiency | Uniform | 0.75–1.25 (±25%) |
| SMPS counting statistics | Normal | σ = 0.2 × √N per size bin |
| Organic aerosol hygroscopicity (κ_OA_) | Uniform | 0.05–0.15 (±50%) |

In each MC draw, the parameters are drawn independently, nf_mPOA_ is recomputed, the SMPS PNSD is perturbed, and PNSD_mPOA_ = nf_mPOA_ × dN/dlogD_p,total_ is integrated to N_mPOA_. CCN concentrations are calculated via κ-Köhler theory (κ = 0.1, pure-water surface tension) at SS = 0.25%, 0.5%, and 1.0%. Results are summarised in Table S3. The MC ensemble confirms that all quantities are robust to the compounded uncertainties: the median values align closely with the deterministic baseline, and the 90% CIs of N_mPOA_ (165–333 cm⁻³), N_CCN_ (e.g., 74–182 cm⁻³ at SS = 0.5%), and the CCN enhancement ratio (0.80–1.89) span ranges that are consistent with the ±30–40% uncertainty estimated in the original manuscript. The 90% intervals reflect the compounding effect of the perturbed parameters through the full analysis chain, while the median values align closely with the deterministic baseline.

Table S3. Monte Carlo uncertainty propagation (N = 1000). Values are baseline (deterministic) and MC median with 90% CI [5th–95th percentile].

| Quantity | Baseline | MC Median [90% CI] |
| --- | --- | --- |
| N_mPOA_ (cm⁻³) | 256 | 257 [165–333] |
| N_CCN_ at SS = 0.25% (cm⁻³) | 57 | 55 [26–105] |
| N_CCN_ at SS = 0.5% (cm⁻³) | 118 | 116 [74–182] |
| N_CCN_ at SS = 1.0% (cm⁻³) | 200 | 198 [130–270] |
| CCN enhancement ratio vs. Gantt2011 (0.5% SS) | 1.34 | 1.30 [0.80–1.89] |
| Pearson r (PNSD_mPOA_ shape vs. baseline) | 1.000 | 0.995 [0.987–0.999] |

Both the MC simulation and the deterministic baseline above assume pure-water surface tension. Surface tension reduction is treated separately in our CCN closure analysis (Fig. S12) because it acts downstream of the PNSD_mPOA_ derivation, affecting only the κ-Köhler activation. The required reduction is constrained by independent CCN measurements and is therefore treated as a separate sensitivity dimension rather than a random perturbation.

Table S4. Density sensitivity analysis for mass-to-number conversion.

| Density (g cm⁻³) | Conversion factor (cm⁻³ per μg m⁻³ POA) | N_mPOA_ scaling relative to nominal |
| --- | --- | --- |
| 1.2 | 169 | 1.17× |
| 1.3 | 156 | 1.08× |
| 1.4 (nominal) | 145 | 1.00× |

This range is narrower than the ±30–40% PNSD uncertainty quantified by the Monte Carlo analysis (Table S3).

Fig. S1. The correlation between bulk PM_1_ concentration of mPOA and mSOA with other chemical components based on AMS measurement. Redish colour represent higher correlation.

Fig. S2 The sea salt contribution (sea salt /(sea salt +mPOA) with growth factor of particles , coloured by the different assumption of κ_POA_. The growth factor was calculated by ZSR mixing role assuming the κ_sea-salt_ of 1.1[68]. This figure indicates that the uncertainty of using the cutoff of 1.4 for POA identification is typically less than 20 %.

Fig. S3. The correlation between the mPOA number fraction (nf_mPOA_) at different sizes with the PM_1_ mass concentration of different chemical species. The highest correlations were found between nf_mPOA_ and mPOA mass concentration, and no correlation was found with BC and MSA, indicating their primary marine sources. Mass concentrations were derived from PM_1_ measurement from AMS.

Fig. S4. Cumulative number fraction of submicron mPOA using PNSD obtained by HTDMA. The lines represent medians, the darker shaded area represents 25^th^ to 75^th^ percentiles, and the lighter shaded area represents 10^th^ to 90^th^ percentiles. Fig. S3 indicates that POA smaller than 60 nm accounted for up to 50% of the total mPOA number concentration.

Fig. S5. The closure calculation between total mPOA volume concentration, derived via HTDMA and AMS, respectively. Blue lines represent regression lines. The electrical mobility measured by SMPS was considered to represent geometric physical aerosol diameter, thus shape factor was ignored. The non-sea-salt volume concentrations were calculated as the sum of nss-SO_4_^2-^, methanesulfonic acid, organic matter, NH_4_^+^, NO_3_^-^, black carbon. The assumed densities were 1.85, 1.48, 1.4, 1.75, 1.72, 1.9, 2.17 g cm^-3^ for nss-SO_4_^2-^, methanesulfonic acid, organic matter, NH_4_^+^, NO_3_^-^, black carbon, and sea-salt, respectively.

Fig. S6. Typical particle number size distribution of polluted air masses (selected by BC > 500 ng m^-3^). The black line represents median values, the shaded area represents 25^th^ to 75^th^ percentiles, and the lighter shaded area represents 10^th^ to 90^th^ percentiles.

Fig. S7. The normalised particle number size distribution of mPOA from using Gantt2011 paramterisation under different wind speed at 10m (U_10_) and sea surface chlorophyll-a concentration.

Fig. S8. Global distribution of simulated surface aerosol mass concentration averaged on December-January-February (DJF) and June-July-August (JJA) of 2019. (a) Sulfate (SO_4_^2-^), (b) sea salt (accumulation and coarse mode) and (c)mPOA mass concentration derived from GEOS-Chem.

Fig. S9. Global distribution of the parameters for AIE estimation averaged on December-January-February (DJF) and June-July-August (JJA) of 2019. (a) downwelling SW at cloud top (F_in_, W·m^-2^), approximated by the CERES EBAF 2.8 downwelling clear-sky shortwave radiation at the surface. (b) cloud albedo, estimated from MODIS cloud optical depth. (c) cloud fraction derived from MODIS.

Fig. S10. Sensitivity of the mPOA particle number size distribution (PNSD_mPOA_) to the event-definition threshold (0.2, 0.3, 0.5, and 1 μg m⁻³). The bimodal shape — Aitken mode at ~60 nm and accumulation mode at ~150 nm — and the relative comparison with the Gantt2011 parameterization remain consistent across all thresholds, confirming the robustness of the derived PNSD_mPOA_.

We have added sensitivity tests with three alternative extrapolation schemes — nf_mPOA_ at 500 nm being the same as at 165 nm, reduced by 5%, and reduced by 10% relative to the 165 nm value — all producing PNSDs with nearly identical shapes, e.g. total N_mPOA_ differed by <4% and CCN at 0.5% differed by <8% across all schemes (Fig. S11). The limited impact of the extrapolation choice on the Gantt2011 comparison arises because the two PNSDs are merged by minimizing the number-weighted residuals for particles larger than 150 nm (Fig. 2c). This alignment anchors the comparison in the >150 nm region where both distributions agree by construction. The key difference between the two PNSDs — the Aitken mode at 30–100 nm — lies in the HTDMA-constrained size range (35–165 nm). The >165 nm extrapolation therefore affects only the tail of both distributions, which are already normalized to each other in this region, making the comparison insensitive to the specific extrapolation scheme.

Fig. S11. Sensitivity of the PNSD_mPOA_ to the >165 nm extrapolation scheme. Three extrapolation schemes are shown (coloured lines), all of which produce nearly identical PNSDs — total N_mPOA_ differs by <4% across schemes. The grey band is the 90% CI from a Monte Carlo ensemble (GF threshold, AMS CE, SMPS counting). Three Gantt2011 curves (grey, varying linetypes) correspond to different organic aerosol densities representing the range of plausible mass-to-number conversion factors. Because the observed PNSD_mPOA_ is scaled to minimise residual across the entire size range — the accumulation-mode tail above 165 nm providing the strongest constraint — neither the choice of extrapolation scheme nor the Gantt2011 density assumption affects the central comparison: the Gantt2011 parameterisation systematically lacks the Aitken mode (peak at ~60 nm) that dominates the observationally-constrained distribution.

During mPOA periods, the CCN activation ratio is consistently high across supersaturations of 0.25–1.0% (Fig. 1d), in line with the strong CCN activity of mPOA reported by Ovadnevaite et al. (2011, *Geophys. Res. Lett.*). To determine whether standard κ-Köhler theory (κ = 0.1, pure-water surface tension σ = 0.0728 N m⁻¹) can reproduce the observed CCN concentrations, we performed an hourly CCN closure analysis during mPOA-dominated summer periods in the clean marine sector (Fig. S12). At each hour, the effective hygroscopicity kappa is computed from the volume-weighted AMS chemical composition (Org, SO4^2-, SeaSalt, NO3-, NH4+, MSA, BC), the critical activation diameter is determined via kappa-Kohler theory, and the measured SMPS PNSD is integrated above D_c_ to predict CCN.

As shown in Fig. S12, CCN concentrations predicted using pure-water surface tension systematically fall below the 1:1 line relative to observations for SS larger than 0.5%, indicating that standard κ-Köhler theory *underestimates* CCN activity during mPOA-dominated periods. To close this gap, we performed an hourly optimization minimizing the difference between predicted and observed CCN/CN, solving for the surface tension reduction required at each supersaturation (Fig. S12b). At SS = 0.5% (representative of marine stratocumulus clouds), the median required reduction is 34% (IQR: −2% to +41%). At SS = 0.25%, the required reduction is 18% (IQR: −12% to +37%). The surface tension reduction is therefore not an arbitrary choice — it is *required* to close the CCN budget for mPOA-dominated aerosol.

Fig. S12. Hourly CCN closure analysis during mPOA-dominated periods (summer, clean marine sector) at Mace Head. At each hour, the effective hygroscopicity κ is computed from the volume-weighted chemical composition measured by AMS (Org, SO₄²⁻, SeaSalt, NO₃⁻, NH₄⁺, MSA, BC), the critical activation diameter is determined via κ-Köhler theory, and the SMPS particle number size distribution at that hour is integrated above D_c_ to predict CCN. (a) Predicted versus observed CCN/CN ratios, assuming pure-water surface tension (σ = 0.0728 N m⁻¹). Points are individual hours; diamonds denote group medians at each supersaturation; the dashed line marks the 1:1 correspondence. Points above the line indicate that surface tension reduction is required for closure. (b) Required surface tension reduction at each supersaturation, derived from hourly optimization minimizing the difference between predicted and observed CCN/CN. Boxes span the interquartile range; whiskers extend to 1.5× IQR. The dashed purple line marks the 30% surface tension reduction assumed in the main analysis. At SS = 0.5% (representative of marine stratocumulus clouds), the median required reduction is 34% (IQR: −2% to +41%), consistent with the 30% conservative assumption.

Fig. S13. Parameter-driven spread in the AIE diagnostic. (a) Distribution of global aerosol indirect effects, ΔAIE, from 108 uncertainty scenarios for each season. Points denote individual scenarios, boxes show the interquartile range, whiskers show the 5th–95th percentile range, and diamonds indicate the baseline case. The vertical jitter of points is used only for visibility. (b) Sensitivity of the global mean cooling magnitude, −ΔAIE, to particle density, hygroscopicity κ, supersaturation SS and surface-tension ratio σ/σ_w_. Values are mean ± 1 s.d. across the remaining uncertainty dimensions at each parameter level. Blue and red indicate DJF and JJA, respectively. The baseline case uses ρ = 1.4 g cm⁻³, κ = 0.10, SS = 0.25% and σ/σ_w_ = 1.0. Error bars represent deterministic scenario spread rather than observational uncertainty or confidence intervals.

ewpage

Fig. S14. Sensitivity of the derived mPOA particle number size distribution (PNSD_mPOA_) to the black carbon threshold used for clean marine sector identification. Results are shown for BC < 10 and BC < 15 ng m⁻³. Shaded bands represent the 90% confidence interval from a 100-iteration Monte Carlo ensemble perturbing the GF threshold, AMS collection efficiency, and SMPS counting statistics. The nearly identical PNSD_mPOA_ shapes and overlapping uncertainty bands confirm that the derived distribution is insensitive to the BC criterion.

## Reference

1.	Liu BYH, Pui DYH, Whitby KT et al. The aerosol mobility chromatograph: a new detector for sulfuric acid aerosols. Atmos. Environ. 1978; 12(1-3): 99–104.

2.	Rader D and McMurry P. Application of the tandem differential mobility analyzer to studies of droplet growth or evaporation. Journal of Aerosol Science 1986; 17(5): 771–87.

3.	Swietlicki E, Hansson HC, Hämeri K et al. [Hygroscopic properties of submicrometer atmospheric aerosol particles measured with H-TDMA instruments in various environments-a review](https://doi.org/10.3402/tellusb.v60i3.16936). Tellus B 2008; 60(3): 432–69.

4.	Tang M, Chan CK, Li YJ et al. [A review of experimental techniques for aerosol hygroscopicity studies](https://doi.org/10.5194/acp-19-12631-2019). Atmos. Chem. Phys. 2019; 19(19): 12631–86.

5.	Xu W, Ovadnevaite J, Fossum KN et al. [Aerosol hygroscopicity and its link to chemical composition in the coastal atmosphere of mace head: Marine and continental air masses](https://doi.org/10.5194/acp-20-3777-2020). Atmos. Chem. Phys. 2020; 20(6): 3777–91.

6.	Białek J, Dall’Osto M, Monahan C et al. [On the contribution of organics to the North East Atlantic aerosol number concentration](https://doi.org/10.1088/1748-9326/7/4/044013). Environ Res Lett 2012; 7(4): 044013.

7.	Gysel M, McFiggans G and Coe H. [Inversion of tandem differential mobility analyser (TDMA) measurements](https://doi.org/10.1016/j.jaerosci.2008.07.013). Journal of Aerosol Science 2009; 40(2): 134 151.

8.	DeCarlo PF, Kimmel JR, Trimborn A et al. [Field-Deployable, High-Resolution, Time-of-Flight Aerosol Mass Spectrometer](https://doi.org/10.1021/ac061249n). Analytical Chemistry 2006; 78(24): 8281 8289.

9.	Ovadnevaite J, Ceburnis D, Canagaratna M et al. [On the effect of wind speed on submicron sea salt mass concentrations and source fluxes](https://doi.org/10.1029/2011jd017379). J Geophys Res Atmos 2012; 117(D16): D16201.

10.	Ovadnevaite J, Ceburnis D, Leinert S et al. [Submicron NE Atlantic marine aerosol chemical composition and abundance: Seasonal trends and air mass categorization](https://doi.org/10.1002/2013jd021330). J Geophys Res Atmos 2014; 119(20): 11850–63.

11.	Middlebrook AM, Bahreini R, Jimenez JL et al. Evaluation of composition-dependent collection efficiencies for the aerodyne aerosol mass spectrometer using field data. Aerosol Sci Technol 2012; 46(3): 258–71.

12.	Petzold A and Schönlinner M. [Multi-angle absorption photometry—a new method for the measurement of aerosol light absorption and atmospheric black carbon](https://doi.org/10.1016/j.jaerosci.2003.09.005). Journal of Aerosol Science 2004; 35(4): 421 441.

13.	Chen B, Lei L, Chevassus E et al. [Differentiation of primary and secondary marine organic aerosol with machine learning](https://doi.org/10.5194/acp-25-14205-2025). Atmos Chem Phys 2025; 25(21): 14205–19.

14.	Ovadnevaitė J, Čeburnis D, Martucci G et al. [Primary marine organic aerosol: A dichotomy of low hygroscopicity and high CCN activity](https://doi.org/10.1029/2011gl048869). Geophys Res Lett 2011; 38(21): L21806.

15.	Prather KA, Bertram TH, Grassian VH et al. [Bringing the ocean into the laboratory to probe the chemical complexity of sea spray aerosol](https://doi.org/10.1073/pnas.1300262110). Proc Natl Acad Sci USA 2013; 110(19): 7550 7555.

16.	Bigg EK and Leck C. [The composition of fragments of bubbles bursting at the ocean surface](https://doi.org/10.1029/2007jd009078). J Geophys Res Atmos 2008; 113(D11): D11209.

17.	Petters MD and Kreidenweis SM. [A single parameter representation of hygroscopic growth and cloud condensation nucleus activity](https://doi.org/10.5194/acp-7-1961-2007). Atmos Chem Phys 2007; 7(8): 1961–71.

18.	van der Werf GR, Randerson JT, Giglio L et al. Global fire emissions estimates during 1997–2016. Earth System Science Data 9(2): 697–720.

19.	Guenther AB, Jiang X, Heald CL et al. The model of emissions of gases and aerosols from nature version 2.1 (MEGAN2.1): An extended and updated framework for modeling biogenic emissions. Geoscientific Model Development 5(6): 1471–92.

20.	Bond TC, Bhardwaj E, Dong R et al. Historical emissions of black and organic carbon aerosol from energy-related combustion, 1850–2000. Global Biogeochemical Cycles 21(2).

21.	Bey I, Jacob DJ, Yantosca RM et al. [Global modeling of tropospheric chemistry with assimilated meteorology: Model description and evaluation](https://doi.org/10.1029/2001JD000807). J Geophys Res Atmos 2001; 106(D19): 23073–95.

22.	Fountoukis C and Nenes A. [ISORROPIA II: a computationally efficient thermodynamic equilibrium model for K+ Ca2+–Mg2+–NH4+–Na+–SO42−–NO3−–Cl−–H2O aerosols](https://doi.org/10.5194/acp-7-4639-2007). Atmos Chem Phys 2007; 7(17): 4639–59.

23.	Gantt B, Meskhidze N, Facchini MC et al. [Wind speed dependent size-resolved parameterization for the organic mass fraction of sea spray aerosol](https://doi.org/10.5194/acp-11-8777-2011). Atmos Chem Phys 2011; 11(16): 8777–90.

24.	Gantt B, Johnson MS, Crippa M et al. [Implementing marine organic aerosols into the GEOS-Chem model](https://doi.org/10.5194/gmd-8-619-2015). Geosci Model Dev 2015; 8(3): 619–29.

25.	Jaeglé L, Quinn PK, Bates TS et al. Global distribution of sea salt aerosols: New constraints from in situ and remote sensing observations. Atmos Chem Phys 11(7): 3137–57.

26.	Croft B, Lohmann U and von Salzen K. Black carbon ageing in the canadian centre for climate modelling and analysis atmospheric general circulation model. Atmos Chem Phys 5(7): 1931–49.

27.	He C, Li Q, Liou KN et al. Microphysics-based black carbon aging in a global CTM: Constraints from HIPPO observations and implications for global black carbon budget. Atmos Chem Phys 16(5): 3077–98.

28.	Kodros JK and Pierce JR. Important global and regional differences in aerosol cloud-albedo effect estimates between simulations with and without prognostic aerosol microphysics. J Geophys Res Atmos 122(7): 4003–18.

29.	Meskhidze N and Nenes A. Phytoplankton and cloudiness in the southern ocean. Science 2006; 314(5804): 1419–23.

30.	Twomey S. Aerosols, clouds and radiation. Atmos Environ Part A 1991; 25(11): 2435–42.

31.	Sun M, Doelling DR, Loeb NG et al. Clouds and the earth’s radiant energy system (CERES) FluxByCldTyp edition 4 data product.

32.	Lacis AA and Hansen J. A parameterization for the absorption of solar radiation in the earth’s atmosphere.

33.	Platnick S, Meyer KG, King MD et al. The MODIS cloud optical and microphysical products: Collection 6 updates and examples from terra and aqua. IEEE Transactions on Geoscience and Remote Sensing 55(1): 502–25.

34.	Sellar AA, Walton J, Jones CG et al. [Implementation of U.K. Earth System Models for CMIP6](https://doi.org/10.1029/2019ms001946). Journal of Advances in Modeling Earth Systems 2020; 12(4).

35.	Zhao X, Liu X, Burrows SM et al. [Effects of marine organic aerosols as sources of immersion-mode ice-nucleating particles on high-latitude mixed-phase clouds](https://doi.org/10.5194/acp-21-2305-2021). Atmos Chem Phys 2021; 21(4): 2305–27.

36.	Gettelman A, Mills MJ, Kinnison DE et al. [The Whole Atmosphere Community Climate Model Version 6 (WACCM6)](https://doi.org/10.1029/2019jd030943). J Geophys Res Atmos 2019; 124(23): 12380–403.

37.	Golaz J, Roekel LPV, Zheng X et al. [The DOE E3SM Model Version 2: Overview of the Physical Model and Initial Model Evaluation](https://doi.org/10.1029/2022ms003156). Journal of Advances in Modeling Earth Systems 2022; 14(12).

38.	Wang H, Easter RC, Zhang R et al. Aerosols in the E3SM version 1: New developments and their impacts on radiative forcing. Journal of Advances in Modeling Earth Systems 2020; 12(1): e2019MS001851.

39.	Burrows SM, Easter RC, Liu X et al. [OCEANFILMS (Organic Compounds from Ecosystems to Aerosols: Natural Films and Interfaces via Langmuir Molecular Surfactants) sea spray organic aerosol emissions – implementation in a global climate model and impacts on clouds](https://doi.org/10.5194/acp-22-5223-2022). Atmos Chem Phys 2022; 22(8): 5223–51.

40.	Gantt B, Johnson MS, Meskhidze N et al. [Model evaluation of marine primary organic aerosol emission schemes](https://doi.org/10.5194/acp-12-8553-2012). Atmos Chem Phys 2012; 12(18): 8553–66.

41.	Seland Ø, Bentsen M, Olivié D et al. [Overview of the Norwegian Earth System Model (NorESM2) and key climate response of CMIP6 DECK, historical, and scenario simulations](https://doi.org/10.5194/gmd-13-6165-2020). Geosci Model Dev 2020; 13(12): 6165–200.

42.	Kirkevåg A, Grini A, Olivié D et al. [A production-tagged aerosol module for Earth system models, OsloAero5.3 – extensions and updates for CAM5.3-Oslo](https://doi.org/10.5194/gmd-11-3945-2018). Geosci Model Dev 2018; 11(10): 3945–82.

43.	Wu T, Zhang F, Zhang J et al. Beijing climate center earth system model version 1 (BCC-ESM1): Model description and evaluation of aerosol simulations. Geosci Model Dev 2020; 13(3): 977–1005.

44.	Séférian R, Nabat P, Michou M et al. [Evaluation of CNRM Earth System Model, CNRM‐ESM2‐1: Role of Earth System Processes in Present‐Day and Future Climate](https://doi.org/10.1029/2019ms001791). Journal of Advances in Modeling Earth Systems 2019; 11(12): 4182–227.

45.	Döscher R, Acosta M, Alessandri A et al. [The EC-Earth3 Earth system model for the Coupled Model Intercomparison Project 6](https://doi.org/10.5194/gmd-15-2973-2022). Geosci Model Dev 2021; 15(7): 2973–3020.

46.	Noije TPC van, Sager PL, Segers AJ et al. [Simulation of tropospheric chemistry and aerosols with the climate model EC-Earth](https://doi.org/10.5194/gmd-7-2435-2014). Geosci Model Dev 2014; 7(5): 2435–75.

47.	Kelley M, Schmidt GA, Nazarenko LS et al. [GISS‐E2.1: Configurations and Climatology](https://doi.org/10.1029/2019ms002025). Journal of Advances in Modeling Earth Systems 2020; 12(8): e2019MS002025.

48.	Bauer SE, Tsigaridis K, Faluvegi G et al. [Historical (1850–2014) Aerosol Evolution and Role on Climate Forcing Using the GISS ModelE2.1 Contribution to CMIP6](https://doi.org/10.1029/2019ms001978). Journal of Advances in Modeling Earth Systems 2020; 12(8).

49.	Boucher O, Servonnat J, Albright AL et al. [Presentation and Evaluation of the IPSL‐CM6A‐LR Climate Model](https://doi.org/10.1029/2019ms002010). Journal of Advances in Modeling Earth Systems 2020; 12(7).

50.	Hauglustaine DA, Balkanski Y and Schulz M. A global model simulation of present and future nitrate aerosols and their direct radiative forcing of climate. Atmos Chem Phys 2014; 14(20): 11031–63.

51.	Hajima T, Watanabe M, Yamamoto A et al. [Development of the MIROC-ES2L Earth system model and the evaluation of biogeochemical processes and feedbacks](https://doi.org/10.5194/gmd-13-2197-2020). Geosci Model Dev 2020; 13(5): 2197–244.

52.	Gutjahr O, Putrasahan D, Lohmann K et al. [Max Planck Institute Earth System Model (MPI-ESM1.2) for the High-Resolution Model Intercomparison Project (HighResMIP)](https://doi.org/10.5194/gmd-12-3241-2019). Geosci Model Dev 2019; 12(7): 3241–81.

53.	Tegen I, Neubauer D, Ferrachat S et al. [The global aerosol–climate model ECHAM6.3–HAM2.3 – Part 1: Aerosol evaluation](https://doi.org/10.5194/gmd-12-1643-2019). Geosci Model Dev 2019; 12(4): 1643–77.

54.	Yukimoto S, Kawai H, Koshiro T et al. The meteorological research institute earth system model version 2.0, MRI-ESM2. 0: Description and basic evaluation of the physical component. Journal of the Meteorological Society of Japan. Ser. II 2019; 97(5): 931–65.

55.	Swart NC, Cole JNS, Kharin VV et al. [The Canadian Earth System Model version 5 (CanESM5.0.3)](https://doi.org/10.5194/gmd-12-4823-2019). Geosci Model Dev 2019; 12(11): 4823–73.

56.	Cole JNS, Salzen K von, Li J et al. [The Canadian Atmospheric Model version 5 (CanAM5.0.3)](https://doi.org/10.5194/gmd-16-5427-2023). Geosci Model Dev 2023; 16(18): 5427–48.

57.	Dunne JP, Horowitz LW, Adcroft AJ et al. [The GFDL Earth System Model Version 4.1 (GFDL‐ESM 4.1): Overall Coupled Model Description and Simulation Characteristics](https://doi.org/10.1029/2019ms002015). Journal of Advances in Modeling Earth Systems 2020; 12(11).

58.	Mahowald NM, Lamarque J, Tie XX et al. [Sea‐salt aerosol response to climate change: Last Glacial Maximum, preindustrial, and doubled carbon dioxide climates](https://doi.org/10.1029/2005jd006459). J Geophys Res Atmos 2006; 111(D5).

59.	Monahan EC. The ocean as a source for atmospheric particles. In: The role of air-sea exchange in geochemical cycling Springer, 1986.

60.	Mårtensson E, Nilsson E, Leeuw G de et al. Laboratory simulations and parameterization of the primary marine aerosol production. J Geophys Res Atmos 2003; 108(D9).

61.	Grythe H, Ström J, Krejci R et al. [A review of sea-spray aerosol source functions using a large global set of sea salt aerosol concentration measurements](https://doi.org/10.5194/acp-14-1277-2014). Atmos Chem Phys 2014; 14(3): 1277–97.

62.	Gong SL. [A parameterization of sea‐salt aerosol source function for sub‐ and super‐micron particles](https://doi.org/10.1029/2003gb002079). Global Biogeochemical Cycles 2003; 17(4): n/a–.

63.	Salter ME, Zieger P, Navarro JCA et al. [An empirically derived inorganic sea spray source function incorporating sea surface temperature](https://doi.org/10.5194/acp-15-11047-2015). Atmos Chem Phys 2015; 15(19): 11047–66.

64.	Smith MH and Harrison NM. [The sea spray generation function](https://doi.org/10.1016/s0021-8502(98)00280-8). Journal of Aerosol Science 1998; 29: S189–90.

65.	Lewis ER and Schwartz SE. Sea Salt Aerosol Production: Mechanisms, Methods, Measurements and Models. 2004.

66.	Burrows SM, Ogunro O, Frossard AA et al. [A physically based framework for modeling the organic fractionation of sea spray aerosol from bubble film Langmuir equilibria](https://doi.org/10.5194/acp-14-13601-2014). Atmos Chem Phys 2014; 14(24): 13601–29.

67.	Vignati E, Facchini MC, Rinaldi M et al. [Global scale emission and distribution of sea-spray aerosol: Sea-salt and organic enrichment](https://doi.org/10.1016/j.atmosenv.2009.11.013). Atmos Environ 2010; 44(5): 670–7.

68.	Zieger P, Väisänen O, Corbin JC et al. [Revising the hygroscopicity of inorganic sea salt particles](https://doi.org/10.1038/ncomms15883). Nature Communications 2017; 8(1).
